# The Loss of HIF1**α** Leads to Increased Susceptibility to Cadmium-Chloride-Induced Toxicity in Mouse Embryonic Fibroblasts

**DOI:** 10.1155/2011/391074

**Published:** 2011-07-17

**Authors:** Ajith Vengellur, Elizabeth Grier, John J. LaPres

**Affiliations:** ^1^Department of Biochemistry and Molecular Biology, Michigan State University, East Lansing, MI 48824-1319, USA; ^2^Center for Mitochondrial Sciences and Medicine, Michigan State University, East Lansing, MI, USA; ^3^Center for Integrative Toxicology, Michigan State University, East Lansing, MI, USA

## Abstract

Wild-type and HIF1*α* −/− MEF cells were used to determine the role of HIF1*α* in cadmium-induced toxicity. Cadmium treatment did not affect HIF1-mediated transcription but led to caspase activation and apoptotic cell death in wild-type and HIF1*α* −/− cells. Cadmium-induced cell death, however, was significantly higher in HIF1*α* −/− cells as compared to their wild-type counterparts. Increased cell death in the HIF1*α* −/− cells was correlated with lower metallothionein protein, elevated levels of reactive oxygen species, and decreased superoxide dismutase enzyme activity. The total and oxidized glutathione levels, and, correspondingly, lipid peroxidation levels were elevated in the null cells compared to wild-type cells, indicating increased antioxidant demand and greater oxidative stress. Overall, the results suggest that basal levels of HIF1*α* play a protective role against cadmium-induced cytotoxicity in mouse embryonic fibroblasts by maintaining metallothionein and antioxidant activity levels.

## 1. Introduction

Most organisms require an aerobic environment for survival and have a well-developed signaling system to adapt to fluctuations in oxygen availability. Hypoxia inducible factor 1*α*  (HIF1*α*) is a protein that plays a role in the cellular and physiological response to decreases in oxygen concentrations (i.e., hypoxia). HIF1*α*  is a multi-domain transcription factor with an oxygen-dependent degradation domain (ODD), a transcriptional activation domain (TAD), and basic Helix-Loop-Helix (bHLH) and Per-Arnt-Sim (PAS) domain [1]. Under normal oxygen or normoxic conditions, HIF1*α*  is constitutively expressed but undergoes rapid proteolysis [2]. During hypoxia, HIF1*α*  escapes degradation, translocates into the nucleus, forms a heterodimer with the aryl hydrocarbon nuclear translocator (ARNT), and regulates the transcription of a large number of target genes by binding to hypoxia response elements (HRE) in their regulatory regions [3–5]. These genes are involved in the adaptive response, including those associated with oxygen transport, vascular development, glycolysis, angiogenesis, and others [6]. 

HIF1*α*  protein function is regulated at the level of protein stability and TAD activity by two different hydroxylases. An asparagine residue within the TAD is modified by the enzyme, Factor Inhibiting HIF1 (FIH), preventing it from interacting with p300 when oxygen is present [7]. HIF1*α*  protein stability is regulated by the prolyl hydroxylase domain containing proteins (PHDs), which modify conserved residues in the ODD of the transcription factor under normoxia. This hydroxylation targets HIF1*α* for ubiquitination via a Von-Hippel-Lindau (VHL) protein-dependent process, and it is subsequently degraded by the 26S proteasome machinery [8–10]. The PHDs are oxygen- and iron-dependent dioxygenases and are inhibited by hypoxia, iron chelators, and certain divalent metals, such as cobalt and nickel [2, 11]. The PHDs contain nonheme iron at their active site and require ascorbate as a cofactor for maintaining iron in the reduced state for catalytic activity [9]. Divalent metals, such as nickel, manganese, and cobalt, can compete for iron at the catalytic site, rendering the enzyme inactive. Though other mechanisms have been proposed for metal-induced hypoxia signaling, it is widely accepted that certain divalent metals are capable of acting as hypoxia mimics in terms of inducing HIF1*α*  stability [9, 11–13]. 

This interplay between metal exposure and the hypoxic signaling pathway suggests a potential role for HIF1*α* in regulating an adaptive response against cadmium-induced oxidative stress. Cadmium is a divalent metal with strong environmental ramifications. Natural processes, such as erosion of rocks, volcanic eruptions, and forest fires, as well as, the burning of fossil fuels and use of sewage sludge as fertilizer, can result in an increased exposure risk to humans. Human exposure results from cigarette smoking and the consumption of contaminated food and water [14]. Exposure to cadmium leads to a broad range of toxicities, including osteoporosis, liver and kidney dysfunction, and cancer. The ability of cadmium to induce this variety of toxicities is most likely related to its ability to produce oxidative stress through the depletion of cellular antioxidant pool, specifically glutathione, and the resulting lipid peroxidation [15–17]. Very little is known, however, about the role of HIF1*α*  in regulating cadmium-induced cell stress.

In the present research, the role of HIF1*α*  in mediating the toxic effects of cadmium exposure was studied using two genetically matched strains of mouse embryonic fibroblasts (MEFs). Our results show that the absence of HIF1*α*  leads to increased susceptibility to cadmium-induced toxicity. Loss of HIF1*α*  alters the cell's ability to maintain the levels of metal transcription factor-1 (MTF-1) and metallothionein, and this decrease correlates with increased cadmium-induced toxicity. The findings also indicate that the basal expression of HIF1*α*  under normoxia plays a crucial role in maintaining the levels of cellular antioxidants such as SOD1, SOD2, and glutathione. Finally, the results suggest that HIF1*α*-mediated cell signaling plays a key role in protecting cells against the oxidative stress produced by cadmium exposure.

## 2. Materials and Methods

### 2.1. Materials

Tissue culture media and supplements were obtained from Invitrogen, Inc. with the exception of cosmic calf serum which was obtained from Hyclone. Oligonucleotides were synthesized by the Macromolecular Facility, Michigan State University, East Lansing, MI. SYBR Green real-time PCR reagents were purchased from Applied Biosystems, CA. All other chemicals were reagent grade and obtained from Sigma Chemical Company, MO.

### 2.2. Cell Culture and Toxicity Assay

Wild-type and HIF1*α* −/− mouse embryonic fibroblast (MEF) cell lines were generated in the lab of Dr. Randall Johnson (UCSD, San Diego) [18]. Briefly, fibroblasts were isolated from conditional null HIF1*α*  mouse embryos. These mice contained loxP sites flanking exon2 of the HIF1*α*  gene. The cells were immortalized and transformed by stable transfection of SV40 large T-antigen and H-ras oncogene, respectively. These immortalized cells were divided, and wild-type and HIF1*α* −/− cells were generated by infecting the cells with adenovirus carrying *β*-galactosidase or cre recombinase, respectively. MEFs were maintained in Dulbeco's Modified Eagle Medium (DMEM) with 10% heat inactivated FBS, penicillin-streptomycin (10 U/mL), nonessential amino acid (0.1 mM), L-glutamine (2 mM) and Hepes Buffer, pH 7.0 (10 mM). Cells were grown in 96 well plates and treated with 0, 1, 2, 5, and 10 *μ*M CdCl_2_ for 72 hours. For ROS scavenger protection studies, cells were treated with CdCl_2_ (5 or 10 *μ*M) alone or in the presence of various reactive oxygen species scavengers, such as reduced glutathione (1 mM), oxidized glutathione (1 mM), melatonin (0.5 mM), N-acetyl cysteine (10 mM), or ascorbic acid (0.5 mM) where indicated. Toxicity assays were performed by replacing the treatment media with 100 *μ*L of media containing 3-(4,5-dimethylthiazolyl-2)-2,5-diphenyltetrazolium bromide (MTT, 0.5 mg/mL), then incubated for 4 hours. Media was then removed by aspiration. The formazan crystals formed were solubilized by adding 200 *μ*L of solvent (1 : 1 DMSO : ethanol 1 : 1 v/v) followed by agitation for 15 minutes. Optical density (OD) measurements were taken at 550 and 630 nm and the difference in OD relative to untreated controls was taken as a measure of cell viability [19].

### 2.3. Protein Extraction and Western Blotting

Wild-type and HIF1*α* −/− cells were grown in normal medium, or in the presence of 150 *μ*M CoCl_2, _or 1, 5 or 10 *μ*M CdCl_2_, or 150 *μ*M CoCl_2_ and 5 *μ*M CdCl_2,_ and protein extracts were prepared. For HIF1*α*  western blot analysis, nuclear protein was extracted as previously described [20]. Briefly, cells were washed twice with cold (4°C) PBS and manually removed by scraping in PBS and pelleted by centrifugation. Cytoplasmic proteins were removed by disrupting the cells in a dounce homogenizer in lysing Buffer A (10 mM Tris (pH7.5), 1.5 mM MgCl_2_, 10 mM KCl, freshly supplemented with 1 mM DTT, 2 mM PMSF, 2 mM Na_3_VO_4_, and 1 protease inhibitor cocktail tablet per 7 mL buffer (MiniTab, Roche)). Nuclei were collected by centrifugation and lysed by resuspension in Buffer C (0.42 M KCl, 20 mM Tris (pH7.5), 20% glycerol, 1.5 mM MgCl_2_, freshly supplemented with 1 mM DTT, 2 mM PMSF, 2 mM Na_3_VO_4_, and 1 protease inhibitor cocktail tablet per 7 mL buffer (MiniTab, Roche)), followed by rotating at 4°C for one hour. Lysate was cleared by centrifugation at 20,000 × g for 20 minutes. For BNIP3, SOD1, SOD2, MT-1/2, and MTF-1 Westerns, total cell lysate was used. Briefly, cells were washed with cold PBS (4°C) and removed from the surface by scraping in cold PBS and collected by centrifugation. Soluble proteins were extracted with cell lysis buffer (25 mM HEPES, pH 7.6, 2 mM EDTA, 10% glycerol, 1 mM PMSF, and protease inhibitor cocktail from Roche) by sonication (5 secs., 4°C). Insoluble material was removed by centrifugation (15,000 × g, 20 min.) and protein concentrations were determined using Bradford assay kit (Bio-Rad) via manufacturer's instructions. An equal amount of protein was separated by SDS-PAGE, and Western blotting was performed with anti-HIF1*α*  or Metallothionein 1/2 (Novus Biologicals, CO., NB-100-479 or NB600-1039) or BNip3 or *β*-actin (Sigma) or SOD1 or SOD2 or MTF-1-specific antibodies (Santa Cruz Biotech, sc-11407 or sc-18503 or H-300) using ECL chemiluminescent detection kit (Amersham Pharmacia). For MT-1/2 a different Western blot procedure was used. Total protein (50 *μ*g) was separated on a 20% SDS-PAGE and transferred to nitrocellulose membrane. After the transfer, the hydrophilic MT-1/2 was immobilized to the membrane by treating with 2.5% glutaraldehyde as previously described [21]. The blots were probed with MT-1/2-specific antibody.

### 2.4. RNA Extraction and Reverse Transcription

RNA extraction was performed using TriZol reagent (Invitrogen) via manufacturer's instructions. Briefly, cells were treated for the specific duration and washed in 1X PBS (4°C). Cells were removed by scraping in the presence of TriZol reagent. Phase separation was accomplished by the addition of chloroform and centrifugation (18,000 × g, 15 min). RNA was precipitated using isopropanol and quantified using a Nano-drop ND-1000 spectrophotometer (Thermo Scientific, Wilmington, DE). Total RNA (1 *μ*g) was reverse-transcribed with Superscript II RNase H- Reverse Transcriptase (Invitrogen) via manufacturer's instructions.

### 2.5. Real-Time Quantitative PCR Analysis

The measurement of BNIP3, SOD1, and SOD2 mRNA levels was performed via real-time PCR technology using ABI SYBR Green core reagent kit. Primers were designed using the web-based application Primer3, biasing towards the 3′ end of the transcript and spanning an intron-exon junction (Table 1) [22]. All of the assays were performed on an ABI 7700 under standard thermal cycling parameters: 95°C for 10 min, 40 cycles of 95°C for 15 seconds, and 60°C for 60 seconds. The murine hypoxanthine guanine phosphoribosyl transferase (HPRT) gene was used as a control and was shown to be unaffected by any treatment used. Standard curves were used to calculate the relative abundance of mRNA from the Ct values.

### 2.6. Determination of ROS Levels

Cells were left untreated or treated with 10 *μ*M CdCl_2_ for 24 hours or 2 mM H_2_O_2_ for 1 hour. Cell were trypsinized, pelleted, and treated with 5 *μ*M CM-H_2_DCFDA (Molecular Probes, OR) for 30 minutes in serum-free media. Cells were washed and resuspended in PBS, supplemented with 10% cosmic calf serum, and analyzed on a BD FACSDiva flow cytometer. Fluorescence intensity, (490 nm excitation and 520 nm emission) of 1 × 10^4^ cells for each sample, was measured and experiments were performed in duplicate.

### 2.7. Determination of Superoxide Dismutase Activity

Total cellular superoxide dismutase activity was measured using superoxide dismutase assay (Cayman Chemicals, MI) as per manufacturer's description. Briefly, treated cells were harvested, pelleted, and lysed by sonication in buffer containing 20 mM Hepes (pH 7.2), 1 mM EGTA, 210 mM mannitol and 70 mM sucrose. Supernatant was collected after centrifuging at 1,500 × g for 5 minutes, and an aliquot was used for performing the superoxide dismutase assay. Enzyme activity was quantified using a standard curve. The results were normalized to total protein levels. 

### 2.8. Determination of Glutathione Levels

Total glutathione levels were determined using a spectrophotometric assay (Cayman Chemicals, Ann Arbor, MI) as per manufacturer's instructions. Briefly, treated cells were harvested by scraping in cold (4°C) PBS and pelleted by centrifugation (1,000 × g, 5 minutes). Cells were sonicated in 1X MES buffer, and insoluble material was removed by centrifugation (5,000 × g, 5 minutes). The supernatant was deproteinated by the addition of an equal volume of 10% metaphosphoric acid and neutralized by adding 0.2 mM triethanolamine. Glutathione concentration was determined by performing a coupled kinetic assay in which the reduction of glutathione is coupled to the conversion of the colorless tetrazolium salt into formazan, then measured spectrophotometrically. The levels of reduced glutathione were determined by treating the samples with 2-vinylpyridine (10 mM) for 1 hour at room temperature before performing the assay. The results were normalized to total protein levels. 

### 2.9. Determination of Lipid Peroxidation of Levels

Malondialdehyde (MDA) levels, indicative of lipid peroxidation, were determined using TBARS assay kit (Cayman Chemicals, Ann Arbor, MI) as per manufacturer's instructions. Briefly, 2 × 10^7^ cells were harvested in PBS after being treated with CdCl_2_ (5 *μ*M) for 24 hours or H_2_O_2_ (2 mM) for 4 hours. Cell extract was prepared by sonication. The samples and standards were reacted with thiobarbituric acid by heating in a boiling water bath for 1 hour, and optical density was measured at 540 nm. The MDA levels in the samples were determined using the standard curve. The results were normalized to total protein levels. 

### 2.10. Statistics

Statistical analysis was performed between treated and untreated samples in each experiment using the *t*-test (two tailed, unequal variance, *P* ≤ 0.05) with the help of Microsoft Excel software.

## 3. Results

### 3.1. HIF1*α*  −/− MEFs Show Greater Susceptibility to CdCl_2_

**Figure 1:**
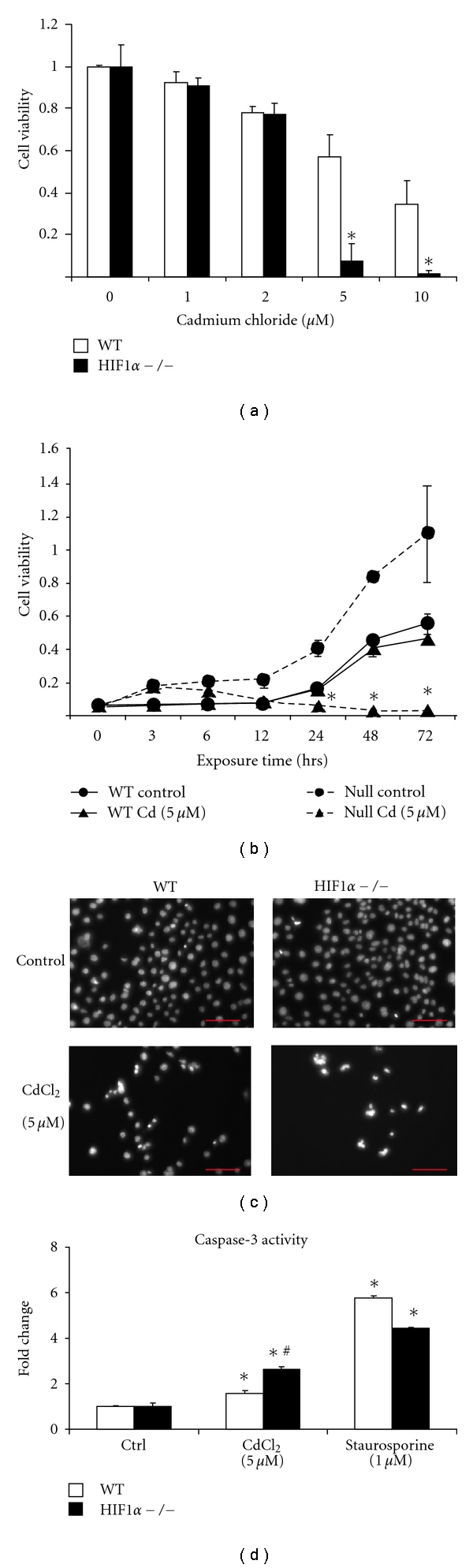
Characterization of cadmium-induced cell death. (a) Wild-type (WT, white bars) and HIF1*α* −/− (black bars) cells were left untreated (0) or exposed to 1, 2, 5, 10 *μ*M CdCl_2_ for 72 hours. Cell viability was assessed using a standard MTT assay. Untreated control values within cell type were set to 1. *P < 0.05 compared to the corresponding controls, *n* = 4. (b) Wild-Type (WT, solid line) and HIF1*α*  −/− (Null, dashed line) were left unexposed (Control, circles) or Cadmium (Cd, 5 *μ*M, triangles) for various times and cell viability was measured using MTT assay. *P < 0.05 compared to the corresponding controls. (c) Wild-type (WT) and HIF1*α* −/− cells were left untreated (Control) or exposed to 5 *μ*M CdCl_2_ for 24 hours and nuclear morphology was observed after staining with Hoechst 33342 dye using fluorescence microscopy. (d) Wild-type (WT, white bars) and HIF1*α* −/− (black bars) cells were left untreated (Ctrl) or exposed to CdCl_2_ (5 *μ*M, 24 hours), or staurosporine (1 *μ*M, 4 hours). Caspase-3 activity was measured using EnZChek caspase-3 assay kit number 2 (Molecular Probes). *P < 0.05 compared to the corresponding controls, *n* = 4. ^#^significant compared to wild type, *P* < 0.05, *n* = 4.

Cadmium-induced cytotoxicity was determined in wild type and HIF1*α*  −/− mouse embryonic fibroblast (MEFs) using an MTT assay. Both cell types were exposed to varying doses of CdCl_2_ (Figure 1(a)) for 72 hours. Wild-type and HIF1*α*  −/− cell lines displayed a dose-dependent decrease in viability following cadmium treatment. Cadmium chloride treatment, however, caused significantly greater cytotoxicity (*P* < 0.05) in the HIF1*α*  −/− cells compared to the wild-type cells at the 5 and 10 *μ*M dose. To verify the timing of cadmium-induced toxicity, WT and HIF1*α*  −/− were exposed to the metal (5 *μ*M) for various times. There was a significant decrease in viability of the HIF1*α*  −/− cells following 24 hours of cadmium (5 *μ*M) as compared to untreated controls (Figure 1(b)). In contrast, there was no significant change in viability in the WT cells following cadmium exposure. 

Previously, we reported that CoCl_2_-induced cytotoxicity correlated with an increase in nuclear condensation; however, this condensation occurred in the absence of caspase-3 activation [20]. Under CdCl_2_ exposure, wild-type and HIF1*α* −/− cells showed significant levels of nuclear condensation, characteristic of apoptosis that appears more pronounced in the null cells (Figure 1(c)). Moreover, HIF1*α*  −/− MEF cells displayed much greater levels of caspase-3 activation following cadmium exposure than the WT cells. In contrast, the positive control, staurosporine (an apoptosis-inducing chemical), induced similar levels of cytotoxicity in the two cell types (Figure 1(d)). These findings indicate that HIF1*α* affects CdCl_2_-induced toxicity in MEFs.

### 3.2. CdCl_2_ Does Not Affect HIF1*α* Protein Levels and Transcriptional Activity in MEFs

**Figure 2:**
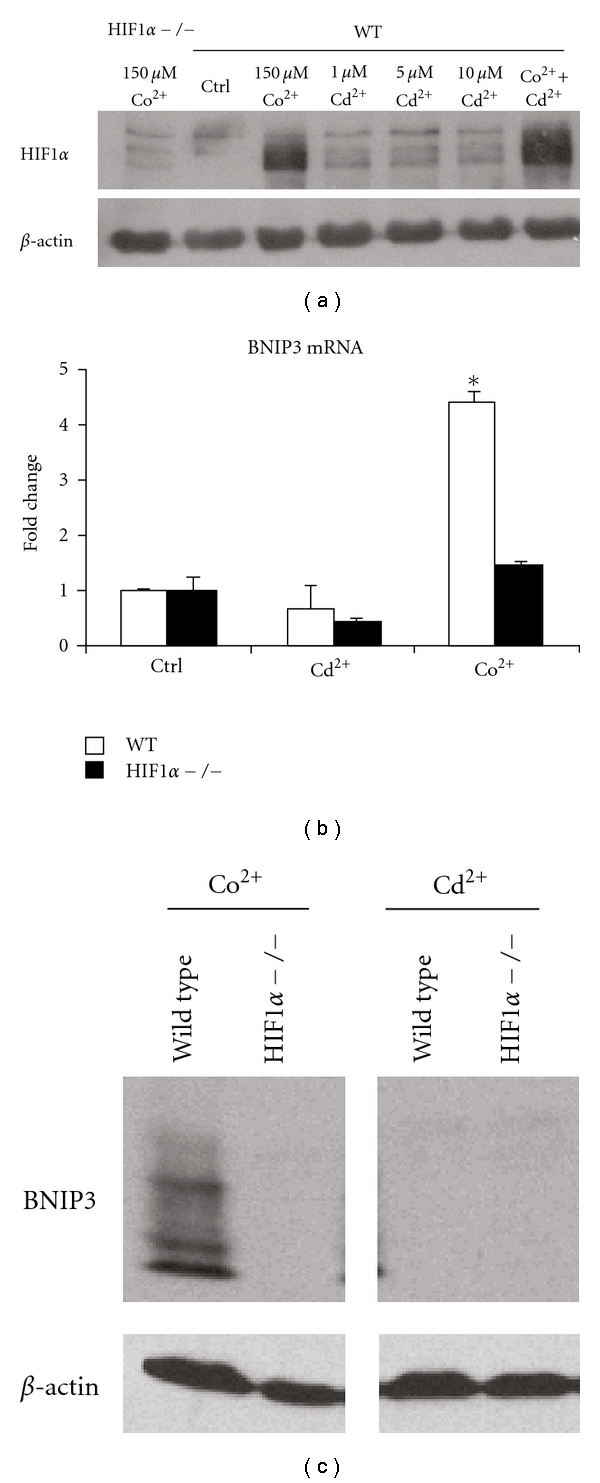
Hypoxia signaling and cytotoxicity to CdCl_2_. (a) Wild-type (WT) cells were left untreated (Ctrl) or exposed to 150 *μ*M CoCl_2_ (Co^2+^), 1, 5 or 10 *μ*M CdCl_2_ (Cd^2+^) or 150 *μ*M CoCl_2_ and 10 *μ*M CdCl_2_ for 24 hours. HIF1*α* −/− cell extract treated with 150 *μ*M CoCl_2_ was used as a negative control. Nuclear protein was extracted and separated by SDS-PAGE, transferred to nitrocellulose membrane and probed with a HIF1*α*  (upper panel) or *β*-actin (lower panel) specific antibody. The bands observed in the cadmium only WT cells are nonspecific as they are also observed in the HIF1*α* −/− cells. (b) BNip3 mRNA expression levels were analyzed by qRT-PCR in wild type (WT, white bars) and HIF1*α* −/− cells (black bars). Cells were left untreated (0), or exposed to 5 *μ*M CdCl_2_ (Cd^2+^) or 150 *μ*M CoCl_2_ (Co^2+^) for 24 hours. Each value was normalized to the control level in the corresponding cell line. *P < 0.05 compared to the corresponding controls, *n* = 4. (c) BNIP3 protein levels were determined in wild type and HIF1*α* −/− cells after treatment with 150 *μ*M CoCl_2_ (Co^2+^) or 5 *μ*M CdCl_2_ (Cd^2+^) for 24 hours using a BNIP3 specific antibody and *β*-actin was used as a loading control (lower Panel).

The effect of cadmium on HIF1*α* protein is cell line specific [23, 24]. Given that HIF1*α*  is primarily regulated at the level of protein stability, the ability of cadmium to stabilize HIF1*α*  in MEFs was tested (Figure 2(a)). Unlike cobalt and nickel, cadmium treatment did not stabilize HIF1*α*  at any dose tested, suggesting the metal was unable to inhibit the PHDs. HIF1*α*  protein levels were detected only in the wild-type cells following CoCl_2_ treatment. In addition, cadmium was unable to alter the levels of HIF1*α*  protein in cells cotreated with cobalt (Figure 2(a)). Previously, our group and others have shown that the mRNA and protein levels of BNip3, a BH3-domain-containing, cell-death-promoting factor, are increased following cobalt chloride treatment in a HIF1*α*-dependent manner [20, 25, 26]. In contrast, the BNip3 mRNA and protein levels were unaffected by cadmium chloride (5 *μ*M) exposure in either cell type (Figures 2(b) and 2(c)). These results confirm our earlier observations and suggest that cadmium-induced cell damage is not regulated by HIF1*α*-mediated increases in BNip3. In addition, the results indicate that the ability of HIF1*α*  to protect against cadmium-induced cytotoxicity in the WT MEFs does not involve changes in HIF1*α*  protein stability. 

### 3.3. HIF1*α* −/− Cells Show Greater Oxidative Stress under Cadmium Treatment

**Figure 3:**
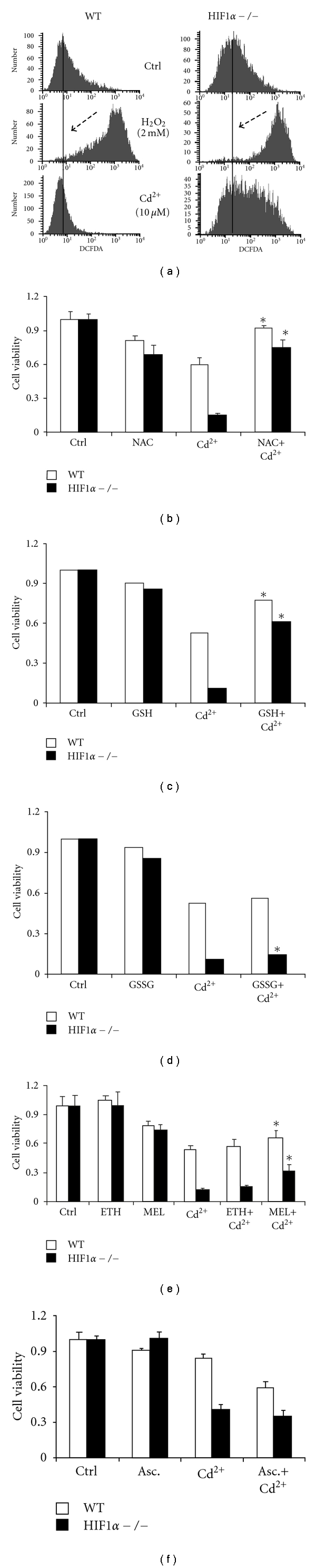
Oxidative Stress in CdCl_2_-mediated cytotoxicity. (a) Wild-type (WT) and HIF1*α*  −/− cells were left untreated (Ctrl), or exposed to 2 mM H_2_O_2_ (H_2_O_2_, 2 hours), or 10 *μ*M CdCl_2_ (Cd^2+^, 24 hours). Reactive oxygen species generated in the cell were measured using the ROS-sensitive dye, CH-H_2_DCFDA, and flow cytometry. Arrow points to the median fluorescence intensity of control cells. (b) Wild-type (white bars) and HIF1*α*  −/− (black bars) cells were treated with 10 *μ*M CdCl_2_ (Cd^2+^) alone or with 10 mM N-acetyl cysteine (NAC) for 24 hours and cell viability was measured using MTT assay. (c)–(f) Wild-type (white bars) and HIF1*α*  −/− (black bars) cells were treated with 5 *μ*M CdCl_2 _(Cd^2+^) alone or with 1 mM reduced glutathione (GSH, C), 1 mM oxidized glutathione (GSSG, D), 0.5 mM Melatonin (MEL, E), 0.2% ethanol (ETH, E) or 0.5 mM ascorbic acid (Asc., F) and cell viability was measured using MTT assay. Control values within cell type were set to 1. *significant compared to cadmium treatment within each cell type, *P* < 0.05, *n* > 4.

Cadmium is known to produce reactive oxygen species (ROS) in various cell types [27]. Hypoxic pre-conditioning and HIF1*α* transcriptional activity can protect against oxidative stress [28–30]. To begin to understand the difference in cadmium sensitivity between the cell lines, we probed the role of HIF1*α* in CdCl_2_-induced ROS levels using CM-H_2_DCFDA. WT cells showed no change in ROS following cadmium challenge. In contrast, exposure to cadmium caused a significant increase in ROS in the HIF1*α* −/− cells as indicated by the shift of median fluorescence intensity to the right of the black line (Figure 3(a)). The lack of a cadmium-induced response in the WT cells was not due to any cell-specific mechanisms, as the positive control, H_2_O_2_, displayed a marked increase in ROS generation in the WT MEFs. Interestingly, the HIF1*α* −/− cells have a higher basal level of ROS compared to wild type cells (Figure 3(a)). This is consistent with the observation that in the absence of HIF1*α*, there is an increased flux through the electron transport chain due to the dysregulation of pyruvate dehydrogenase kinase, leading to elevated levels of ROS [31].

To determine if the increased levels of ROS in the HIF1*α*  −/− cells play a role in their increased sensitivity to cadmium-induced cytotoxicity, cell viability assays were performed in the presence of various scavengers. N-acetyl cysteine (NAC), a known antioxidant, acts as a precursor for glutathione and can protect cells against oxidative injury caused by metals and other agents [32]. NAC prevented metal-induced cell damage in both WT cells and HIF1*α*  −/− MEF cells (Figure 3(b)). Given NAC's ability to chelate heavy metals, such as cadmium, other antioxidants were also tested. In both cell types, reduced glutathione offered protection from cadmium-induced reduction in viability (Figure 3(c)). The level of protection was much more modest, but significant, in the WT cells. Oxidized glutathione did not protect the wild-type cells, however, there was a slight but statistically significant protection in the null cells (Figure 3(d)). Melatonin, a natural antioxidant, also protected both cell types against cadmium treatment, although to a lesser degree (Figure 3(e)). Interestingly, ascorbic acid did not protect either cell type against cadmium-mediated cell injury (Figure 3(f)). These results point to the roles of increased ROS and oxidative stress as explanations for the increased cadmium-induced cytotoxicity in HIF1*α*  −/− MEFs. 

### 3.4. HIF1*α* Affects Metallothionein and Metal Transcription Factor-1 Protein Levels upon CdCl_2_ Exposure

**Figure 4:**
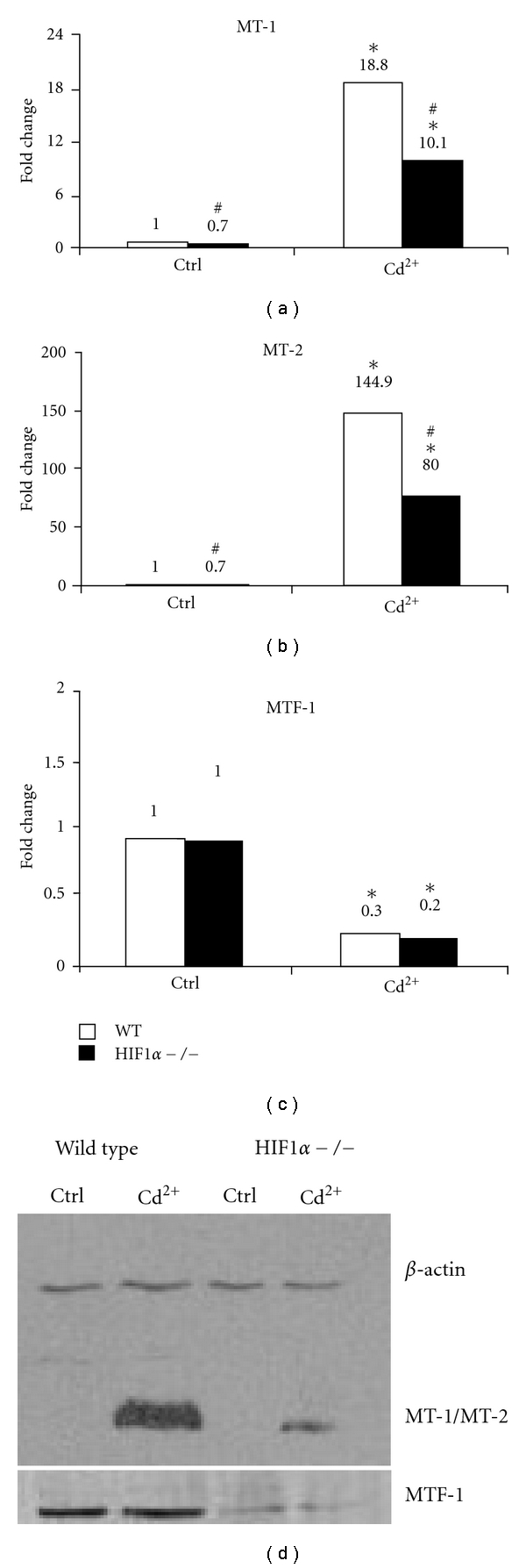
Cadmium-induced changes in metallothionein and MTF-1. Wild-type (WT, white bars) and HIF1*α*  −/− (black bars) cells were left untreated (Ctrl) or 5 *μ*M CdCl_2_ (Cd^2+^) for 24 hours. Metallothionein-1 (A, MT-1), Metallothionein-2 (B, MT-2), and Metal Transcription Factor-1 (C, MTF-1) mRNA levels were measured using qRT-PCR. All values were normalized to wild-type control. The number above each bar is the fold change for that particular treatment. *P < 0.05 compared to respective controls, ^#^ *P* < 0.05 compared to wild-type cells within treatment group, *n* = 6. G. Metallothionein-1/2 (middle panel) and MTF-1 (lower panel) protein levels were determined in wild-type and HIF1*α*  −/− cells after treatment with 5 *μ*M CdCl_2_ (Cd^2+^) for 24 hours. *β*-actin was used as a loading control (upper panel).

Given metallothioneins' role as transporters of bioavailable cadmium and zinc, it was important to determine if changes in their levels could explain the difference in cadmium sensitivity between the two cell types [33, 34]. Metallothioneins have also been shown to be induced by cadmium as an adaptive response to protect against toxicity from the metal [35, 36]. It is also known that hypoxia induces the expression of metallothionein through its ability to induce Metal Transcription Factor-1 (MTF-1) binding to metal response elements [37, 38]. We measured the mRNA levels of Metallothionein-1 (MT-1), Metallothionein-2 (MT-2), and MTF1. Both cell types showed a robust increase in MT-1 and MT-2 expression upon cadmium exposure (Figures 4(a) and 4(b)). Even though the cadmium treatment induced MT-1 and MT-2 in both cell types, there was a significant 30 and 45% reduction in the mRNA levels of MT-1 and MT-2 in the control and cadmium-treated samples of the HIF1*α*  −/− cells compared to wild-type cells, respectively (Figures 4(a) and 4(b)). Cadmium treatment reduced MTF-1 mRNA levels in wild-type and HIF1*α*  −/− cells (Figure 4(c)). We also measured the protein levels of MT-1/2 (the antibody could not distinguish between the two isoforms) and MTF-1. Wild-type and HIF1*α*  −/− cells had undetectable levels of MT-1/2 protein in the control samples (Figure 4(d), middle panel). CdCl_2_ exposure led to a large increase in metallothionein protein in WT cells. There was also an induction of metallothionein protein in the HIF1*α*  −/− cells following cadmium challenge, however, this increase was much less than that of the WT cells (Figure 4(d)). The decreased MT-1/2 protein expression in the HIF1*α*  −/− compared to WT metal-treated cells confirm the decreased mRNA levels (Figures 4(a), 4(b), and 4(d)). MTF-1 protein levels were also markedly greater in the wild-type cells compared to HIF1*α*  −/− cells in both control and cadmium-treated samples (Figure 4(d), lower panel). The protein levels, however, did not change between controls and treated samples in either cell type. The above results indicate that HIF1*α* plays a major role in maintaining the basal levels of MTF-1 and in regulating the cadmium-induced metallothionein protein levels and might indicate a possible mechanism for the increased ROS and cadmium-induced cytotoxicity in HIF1*α*  −/− cells.

### 3.5. Superoxide Dismutase Levels in CdCl_2_-Treated Cells

**Figure 5:**
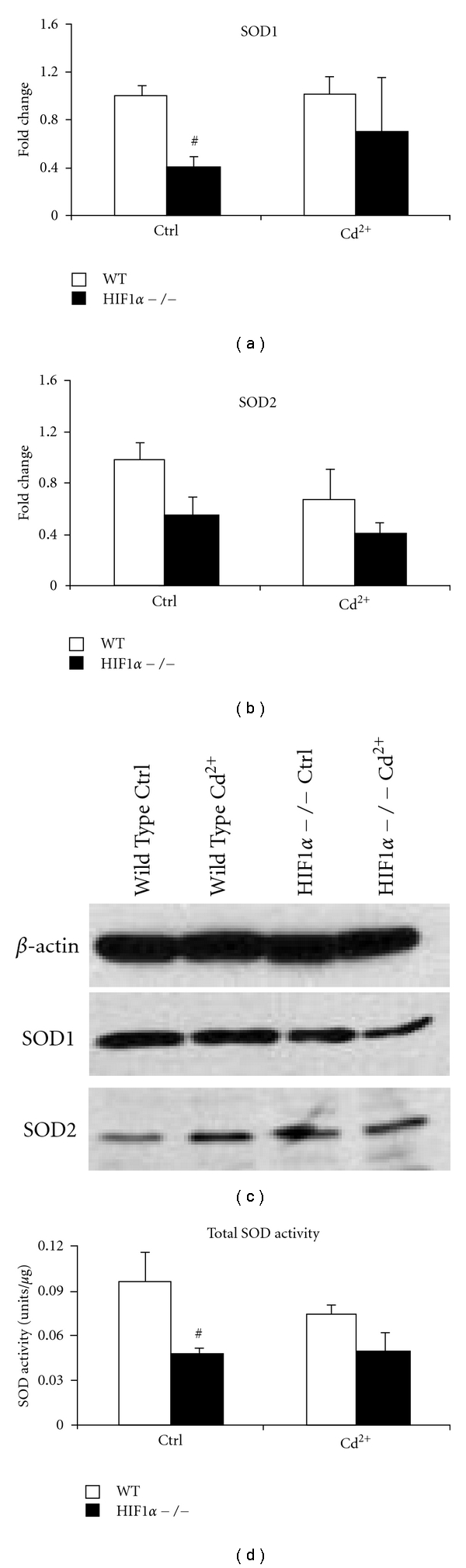
Expression and activity of superoxide dismutases. Transcript levels of SOD1 (a) and SOD2 (b) were determined using qRT-PCR in wild-type (WT, white bars) and HIF1*α*  −/− cells (black bars). Each value was normalized to the wild-type control levels. Cells were left untreated (Ctrl) or treated with 5 *μ*M CdCl_2_ (Cd^2+^) for 24 hours. (c) Protein levels of both SOD1 (middle panel) and SOD2 (bottom panel) were determined by Western blotting in wild-type (WT) and HIF1*α*  −/− cells. *β*-actin levels (upper panel) were used as a loading control. Cells were treated as in (a) and (b). (d) Total superoxide dismutase enzyme activity in wild-type (WT, white bars) and HIF1*α*  −/− (black bars) cells were determined. Cells were treated as in (a) and (b). ^#^significant compared to wild-type cells, *P* < 0.05, *n* = 4.

CdCl_2_ exposure has been shown to produce superoxides [39]. Superoxide is rapidly removed from the cellular space by superoxide dismutases (SODs), which convert the superoxide into oxygen and hydrogen peroxides [40]. To determine if cadmium-mediated alterations in ROS are correlated with changes in SOD expression, the mRNA levels of SOD1 and SOD2 were analyzed by qRT-PCR. In agreement with our previously published report, SOD1 and SOD2 mRNA levels were lower in the untreated HIF1*α*  −/− cells compared to WT cells (Figures 5(a) and 5(b)) [6]. CdCl_2 _treatment did not alter the expression of SOD1 or SOD2 in either cell type. To determine if these changes in mRNA expression correlated with changes in SOD protein levels, SOD1 and SOD2 were analyzed by Western blot analysis. In agreement with the mRNA data, SOD1 protein levels were slightly lower in the HIF1*α*  −/− control cells compared to wild-type cells. Metal exposure did not alter the levels of SOD1 in the wild-type cells, whereas a reduction was observed in HIF1*α*  −/− cells (Figure 5(c)). Basal level of SOD2 protein was marginally higher in the HIF1*α*  −/− cells compared to WT cells. CdCl_2_ treatment caused a slight increase in SOD2 protein levels in wild-type cells and there was no change in the HIF1*α*  −/− cells. Finally, total superoxide dismutase activity was also consistently lower in HIF1*α*  −/− cells compared to WT cells (Figure 5(d)). These results suggest that HIF1*α*  −/− cells have a reduced capacity to cope with superoxide stress compared to their WT counterparts and this might explain the difference in ROS levels and susceptibility to cadmium-induced cytotoxicity.

### 3.6. Effect on Glutathione Levels in Cells Treated with CdCl_2_

**Figure 6:**
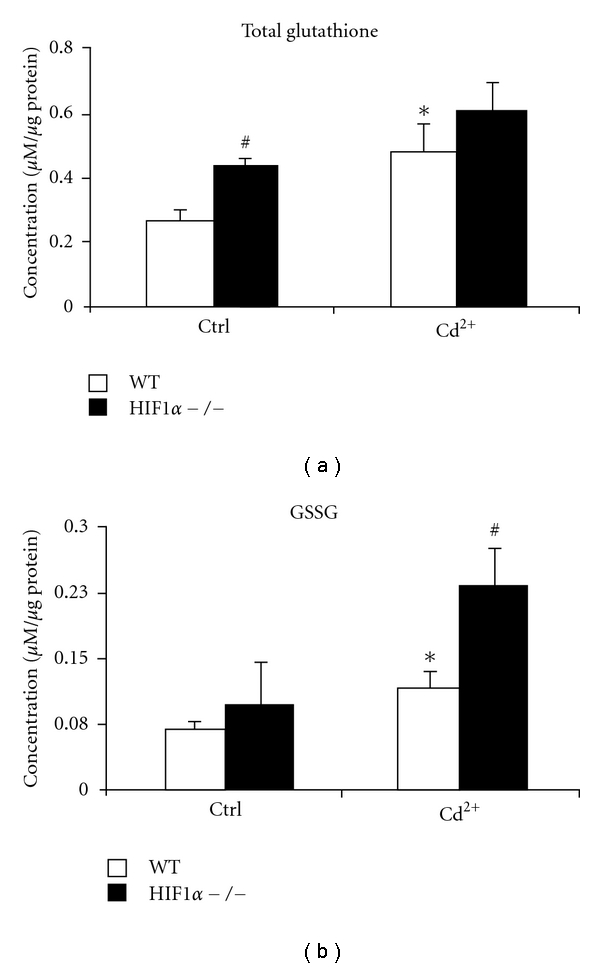
Cellular glutathione levels under CdCl_2_ treatment. Total cellular glutathione levels (a) and reduced glutathione levels (b) in wild-type (WT, white bars) and HIF1*α*  −/− (black bars) cells were determined. Cells were left untreated (Ctrl), or treated with 5 *μ*M CdCl_2_ (Cd^2+^) for 24 hours. *P < 0.05 compared to respective controls, *n* = 4. ^#^ *P* < 0.05, significant compared to wild-type cells, *n* = 4.

Glutathione plays a critical role in various detoxification reactions within the cell by acting as a reducing agent [41, 42]. Cadmium is known to deplete the cellular glutathione pool by covalently binding to its sulfhydryl groups and thereby compromises the cell's ability to protect against oxidative stress [17]. To determine if metal exposure can alter the glutathione pool within the wild-type or HIF1*α*  −/− cells, total cellular glutathione was assessed [43]. HIF1*α*  −/− cells showed higher total glutathione levels under control conditions compared to WT cells (Figure 6(a)). Both cell types showed an increase in total glutathione following cadmium exposure but the increase was statistically significant in the wild-type cells only. The absolute levels of total glutathione remained higher in the HIF1*α*  −/− cells under control and cadmium treatment conditions. This may be an indication of the greater demand for antioxidants in the HIF1*α*  −/− cells due to increased oxidative stress in the control and CdCl_2_ treatment conditions. 

Cells under oxidative stress utilize glutathione (GSH) to reduce the biological molecules to their native state and in the process GSH is converted into its oxidized form, glutathione disulfide (GSSG). The GSSG levels were elevated in both cell types following cadmium exposure (Figure 6(b)). But the levels were markedly higher in the HIF1*α*  −/− cells compared to the wild-type cells (Figure 6(b)). This indicates that the ratio of reduced glutathione to total glutathione is much lower in the HIF1*α*  −/− cells under CdCl_2_ treatment compared to wild-type cells. This decreased level of reduced glutathione will compromise the cell's ability to cope with oxidative damages to the cellular organelles and biomolecules (Figures 6(a) and 6(b)). 

### 3.7. Elevated Lipid Peroxidation in HIF1*α*  −/− Cells Treated with CdCl_2_

**Figure 7:**
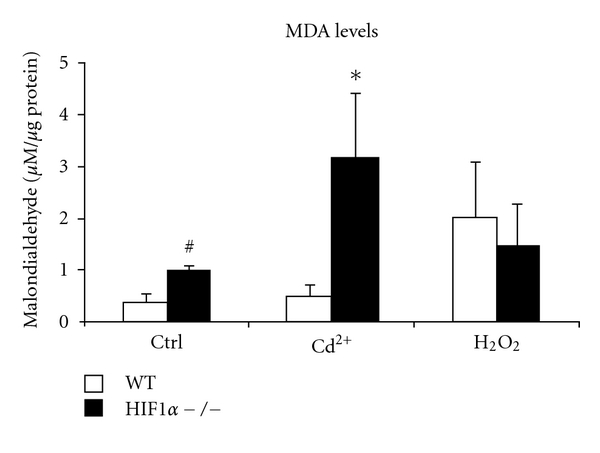
Lipid peroxidation under CdCl_2_ treatment. Lipid peroxidation levels were determined in wild type (WT, white bars) and HIF1*α*  −/− (black bars) by measuring malondialdehyde (MDA) levels. Cells were left untreated (Ctrl), or treated with 5 *μ*M CdCl_2_ (Cd^2+^) for 24 hours or 2 mM H_2_O_2_ for 4 hours. *P < 0.05 compared to respective controls, *n* = 4. ^#^ *P* < 0.05, significant compared to wild-type cells, *n* = 4.

Cadmium is known to induce lipid peroxidation in cells [27, 44]. The cellular consequence of the increased ROS levels and the decreased scavenging capacity was determined by analyzing the levels of lipid peroxidation of the two cell types in the presence and absence of cadmium. One measure of lipid peroxidation is the production of malondialdehyde (MDA) which is formed by the decomposition of lipid hydroperoxides of polyunsaturated fatty acids. MDA levels were elevated in the HIF1*α*  −/− control cells compared to the wild-type cells (Figure 7). This is in agreement with the increased ROS levels in the null cells (Figure 3(a)) and decreased SOD activity (Figure 5(d)). Most interestingly, the MDA levels were extremely high in the CdCl_2_-treated HIF1*α*  −/− cells, suggesting massive lipid peroxidation and resulting damage to biomolecules. The hydrogen peroxide-treated cells showed elevated MDA levels in both cell types (Figure 6). Taken in sum, the results indicate that HIF1*α* plays a major role in protecting cells against cadmium-induced oxidative stress.

## 4. Discussion

Cell signaling pathways in eukaryotic cells were developed through evolution for meeting cellular needs. It is not uncommon to see a single signaling pathway participating in disparate biological processes. The evolution of hypoxia signaling pathways to mediate a response to hypoxia was the result of transition from an anaerobic to an aerobic environment. This change in environment facilitated the development of an electron transport chain, increasing the cellular energy production. This oxygen-dependent energy production, however, increased the susceptibility to oxidative damage, through the production of ROS. It is not surprising that nature evolved a signaling system that has a dual role of maintaining energy levels following loss of oxygen tension, as well as protection against oxidative stress such as the hypoxia signaling pathway. HIF1 plays a central role in maintaining ATP levels following loss of oxygen tension by increasing the glycolytic capacity of the cells [45]. HIF1 also regulates the expression of various genes involved in protecting cells against oxidative stress. Hypoxia induces the expression of erythropoietin, NQO1, HO-1, heat shock proteins, and other factors that are involved in the cell survival as well as detoxification of ROS [46–48]. HO-1 and NQO-1 are also induced by heavy metal exposure and are thought to play a protective role in metal induced toxicity [49].

In this study, we investigated the role of HIF1*α*  in modulating cadmium-induced cytotoxicity. The results demonstrate that HIF1*α*  −/− cells are more sensitive to cadmium-induced toxicity. This is in contrast to the cobalt-induced toxicity, which is more toxic to wild-type MEFs. Presumably, this difference is due, in part, to each metal's ability to inhibit PHDs and thus regulate HIF1*α*  stability [20]. Cobalt is capable of stabilizing HIF1*α*  and it promotes HIF1-mediated transcription. In contrast, cadmium treatment does not inhibit the PHDs and lead to HIF1*α*  stabilization nor was it able to drive the expression of hypoxia-regulated genes. These results are in agreement with previously published reports [23]. Cadmium was also unable to inhibit cobalt-induced HIF1*α*  stabilization. This is in contrast to the result observed in Hep3B cells where cadmium was able to inhibit hypoxia-induced HIF1*α* stabilization [24]. This disparity is probably due to the different treatments and cell types. Under cadmium exposure, wild-type and HIF1*α*  −/− cells undergo apoptosis, characterized by caspase activation and chromatin condensation, independent of BNIP3 expression. Interestingly, cadmium-induced changes in caspase-3 activity and nuclear condensation were more pronounced in the HIF1*α*  −/− cells. 

Metallothioneins are the major transporters and storage proteins of cadmium in cells. HIF1*α* and metallothioneins have been shown to have a reciprocal relationship. Metallothionein overexpression has been shown to influence HIF signaling and HIF1*α* plays a role in the expression of metallothioneins, especially under hypoxia [37, 38]. Our group has shown that basal MT-1 mRNA levels are dependent on HIF1*α* in MEFs (Supplementary Table H in [6]). The partial dependence of metallothionein mRNA and protein on HIF1*α* may be due to changes in MTF1 or other unknown metallothionein regulatory protein(s) whose expression is influenced by HIF1*α*. For example, the basic helix-loop-helix-leucine zipper protein, USF (upstream stimulatory factor family), plays a dominant role on induction of metallothionein transcription following cadmium and H_2_O_2_ exposure [50]. USF associates with an unknown protein that binds to an antioxidant response element that overlaps with the USF-binding site (USF/ARE), in the mouse MT-1 promoter. If USF or its partner protein is influenced by HIF1*α* levels is unknown [50]. There is evidence for a cooperative role of HIF1*α*  and USF in mediating gene regulation. In primary rat hepatocytes, hypoxic induction of glucokinase is mediated by binding of HIF1*α* and USF-2a to a common binding site in the promoter region [51]. There is also evidence for MTF-1-independent induction of metallothionein by cadmium exposure. In IMR-32 human neuroblastoma cells, cadmium has been shown to induce MT-1 expression independent of MTF-1 and zinc [52]. We also show that MTF1 protein expression is influenced by HIF1*α* even though their levels do not change upon cadmium treatment (Figure 4(d)). Whether this is a direct or indirect response is not clear. MTF1 is known to be regulated at the posttranslational level by cytoplasmic-nuclear trafficking which is supposed to involve other signal transduction pathways such as kinases and phosphatases [53, 54]. This might partly explain the discordance in mRNA and protein levels of MTF1 and metallothionein levels in these cells.

Another important characteristic of cadmium exposure was increased ROS levels in the HIF1*α*  −/− cells compared to wild-type cells. Redox active metals such as iron, cobalt, and nickel are thought to produce ROS through Fenton-like reactions. In contrast, redox inactive metals such as mercury and cadmium are thought to deplete cellular sulfhydryl antioxidant reserves and interfere with cellular antioxidant enzymes, such as glutathione transferases and glutathione peroxidases, leading to oxidative stress [17, 55]. In these experiments, cadmium was capable of producing much higher ROS levels in HIF1*α*  −/− cells. Interestingly, untreated HIF1*α*  −/− cells had a much higher level of ROS compared to the wild-type cells (Figure 3(a)). Similar results in MEF cells were observed by a number of groups, including Kim et al., who found an increased production of H_2_O_2_ in HIF1*α*  −/− cells compared to wild-type MEFs [31]. Their data suggest that HIF1*α*  −/− MEF cells have lower levels of pyruvate dehydrogenase kinase1 (PDK1), a key inhibitory enzyme for the pyruvate dehydrogenase complex. This complex regulates the ability of pyruvate to enter the mitochondria. The authors conclude that increased carbon flux through the tricarboxylic acid cycle (TCA cycle), due to loss of PDH inhibition and subsequent electron flow through the ETC in HIF1*α*  −/− cells, leads to increased oxidative stress. Our data also suggest that the most effective method to counter cadmium toxicity is to increase the glutathione reserves by treating with reduced glutathione or N-acetyl cysteine (NAC), a precursor for glutathione, which is consistent with many other studies [56, 57]. Melatonin, which has been shown to help in the maintenance of cellular reduced glutathione pool as well as modulate the cadmium-induced expression of metallothionein-2a, was also capable of partially protecting the cells against cadmium insult [58, 59]. These results are consistent with the observations of Im et al. [56]. They saw that in primary cortical glia cell cultures NAC and reduced glutathione protected against cadmium-mediated injury whereas ascorbate or oxidized glutathione did not, indicating the role of glutathione depletion in causing the injury. 

Oxidative stress can be a result of reduced elimination of reactive species. Previous reports have demonstrated that HIF1*α*  −/− cells have a lower level of SOD1 and SOD2 mRNA compared to wild-type cells [6] (Figures 5(a) and 5(b)). Overall levels of SOD1 protein and total superoxide dismutase activity were also low in the HIF1*α*  −/− cells compared to their wild-type counterparts. This decrease in dismutase activity might bias the HIF1*α* null cells towards cadmium-induced cytotoxicity. SOD1 is transcriptionally regulated by the binding of NF-kB, AP-1, AP-2, C/EBP*α*, SP1, EGR1, and WT1 proteins [60, 61]. NF-kB is a major transcription factor that regulates the constitutive and inducible expression of SOD1 and SOD2 [60]. NF-kB activity is modulated by HIF prolyl hydroxylase enzymes which are targets of HIF1*α*-mediated transcription [62]. Also, SOD2 has been demonstrated to be a HIF2 target gene and HIF2*α*  knockout mice have decreased SOD2 levels coinciding with increased cellular ROS levels [63, 64]. It is not known if HIF1*α*, the major isoform in MEFs, plays a similar role in maintaining SOD2 levels. CdCl_2_-treatment did not significantly alter the expression of SOD1 in either cell type. There was a minor reduction in protein levels in the CdCl_2_ treated HIF1*α*  −/− cells compared to untreated controls. This is in contrast with the reports of increased expression of SOD1 by heavy metals in transient transfection experiments [65]. However, Thijssen et al. reported reduced levels of SOD1 in rats exposed to chronic low dose exposure to cadmium [49]. Another group has reported an increase in the nuclear activity of SOD upon exposure to cadmium in a lymphocytic cell line, whereas we saw a decrease in activity in the wild-type cells [66]. In an *in vivo* rat study, Shukla et al. have observed strong inhibitory effect on SOD activity by cadmium exposure [67]. It is possible that cell type, dose, and model system may determine the modulation of SODs upon cadmium exposure. In the present study, it is difficult to separate the effects of changes in mRNA and protein expression, and cadmium-induced SOD activity inhibition, except to say that HIF1*α*  seems to play a crucial role in maintaining total superoxide dismutase activity.

Another mechanism cells use to cope with oxidative stress is the glutathione system. In these experiments, the cellular glutathione pool was increased in wild-type and HIF1*α*  −/− cells under CdCl_2_ treatment. The cadmium-induced increase in available glutathione was only significant in the WT cells. This lack of metal-induced increase in glutathione might play a role in the cadmium cytotoxicity in the null cells. The higher glutathione levels in the HIF1*α*  −/− cells compared to wild-type cells, in all treatments studied, may suggest an increased demand for glutathione for a cell under oxidative stress as indicated by elevated levels of GSSG (Figure 6). A reduction in the level and activity of glutathione utilizing enzymes may also result in an increased cellular glutathione pool. We have shown earlier that mRNA levels of GST*μ*1, a major mitochondrial xenobiotic enzyme, is significantly lower in the HIF1*α*  −/− cells compared to wild-type cells [6]. The expression levels of GST*α*1, another glutathione transferase enzyme, were also lower in the HIF1*α*  −/− cells (data not shown). This may also have consequences in the reactive oxidation species scavenging ability of the cells. The end result of increased oxidative stress is biomolecular damage. The elevated MDA levels in the HIF1*α*  −/− cells (Figure 7) are evidence for their inability to cope with increased oxidative stress under CdCl_2_ exposure.

Taken together, our results suggest that the adaptive response of the HIF1 signaling pathway through the induction of metallothioneins, and maintenance of a functional and potent ROS scavenging system, protects cells against cadmium insult. These same factors may play a similar role in protection against other ROS-producing agents such as heavy metals or other compounds. This study underscores the significant role played by HIF1*α*  in the presence of oxygen and its role in maintaining cellular homeostasis. 

## Figures and Tables

**Table 1:** qRT-PCR primers.

Gene	Accession	Forward	Reverse
HPRT	NM_013556	AAGCCTAAGATGAGCGCAAG	TTACTAGGCAGATGGCCACA
BNip3	NM_009760	GGCGTCTGACAACTTCCACT	AACACCCAAGGACCATGCTA
SOD1	BC002066	GAGACCTGGGCAATGTGACT	TTGTTTCTCATGGACCACCA
SOD2	BC018173	AACTCAGGTCGCTCTTCAGC	GCTTGATAGCCTCCAGCAAC
MT-1	NM_013602.3	CACCAGATCTCGGAA TGGAC	AGGAGCAGCAGCTCT TCTTG
MT-2	NM_008630.2	TGCTCCTGTGCCTCCGATGG	AGCACTTCGCACAGCCCACG
MTF-1	NM_008636.4	CCAACTCCTAACACGGCAAT	TTCGATGCTTGCTGAATTTG
